# Enhancing activity of β-lactam and fluoroquinolones antibiotics by artemisinin and its derivatives against MDR *Escherichia coli*

**DOI:** 10.3389/fvets.2022.1048531

**Published:** 2022-11-10

**Authors:** Shahbaz Ul Haq, Ling Wang, Wenzhou Guo, Amjad Islam Aqib, Afshan Muneer, Muhammad Saqib, Saad Ahmad, Muzafar Ghafoor, Amir Iftikhar, Keyuan Chen, Jianping Liang

**Affiliations:** ^1^Key Laboratory of New Animal Drug Project, Gansu Province, Key Laboratory of Veterinary Pharmaceutical Development, Ministry of Agriculture and Rural Affairs, Lanzhou Institute of Husbandry and Pharmaceutical Sciences of Chinese Academy of Agriculture Sciences, Lanzhou, China; ^2^Department of Medicine, Cholistan University of Veterinary and Animal Sciences, Bahawalpur, Pakistan; ^3^Department of Zoology, Cholistan University of Veterinary and Animal Sciences, Bahawalpur, Pakistan; ^4^Department of Clinical Medicine and Surgery, University of Agriculture, Faisalabad, Pakistan; ^5^Department of Clinical Medicine and Surgery, Faculty of Veterinary and Animal Sciences, The Islamia University of Bahawalpur, Bahawalpur, Pakistan

**Keywords:** multidrug resistance *E. coli*, beta lactam, fluoroquinolones, artemisinin, fractional inhibitory concentration index

## Abstract

Artemisinin and its derivatives had played a biocidal role in biomedical remedies, while they were expected to enhance the activity of antibiotics against multiple drug-resistant (MDR) bacteria. The current study evaluated the interaction of artemisinin (ART), dihydroartemisinin (DHA), artesunate (AS), and artemisinic acid (AA) with β-lactam and fluoroquinolones antibiotics against *Escherichia coli*. Antibiotic strip test (*E*-test), Kirby Bauer's disc test (KB method), and broth microdilution method were adopted for susceptibility analysis, while the checkerboard method was applied to assess synergisms. ART, DHA, AS, and AA showed significantly enhanced antibacterial effects of β-lactam antibiotics against different strains of *E. coli*. The study showed ciprofloxacin to be most effective by presenting the least MIC (0.017125 ± 0.0022 μg/ml), while oxacillin was least effective (MIC 256 μg/ml) against *E. coli*. Synergism between AA and penicillin G (75%), ampicillin (25%), and oxacillin (50%) was observed in all isolates tested. AA and AS significantly decreased the MIC of ampicillin (−0.912 ± 0.908 μg/ml) and ciprofloxacin (−0.901 ± 0.893 g/ml), respectively. Artemisinin and its derivatives increased antibiotic accumulation within *E. coli* in a dose-dependent manner. The time-kill assay significantly reduced the bacterial number within 24 h of incubation. The study thus concludes greater room for improvement in enhancing the efficacy of antibiotics if used with artemisinin and its derivatives.

## Introduction

*Escherichia coli, Salmonella* species, and *Staphylococcus aureus* have affected poultry, dairy animals, and pets because they are resistant to a broader range of antibiotics (1–4). Among gram-positive bacteria, a rise in antimicrobial resistance has resulted in novel strains of *S. aureus* like methicillin-resistant *S. aureus*, vancomycin-resistant *S. aureus* (5, 6), and vancomycin-intermediate *S. aureus*. On the other hand, among gram-negative bacteria, *E. coli* is the top prevalent bacteria with extended resistance patterns. These bacteria possess β-lactamases of four classes, i.e., serine penicillinases, Metallo-β-lactamases, cephalosporinases, and oxacillinases (7). China has been found to possess a higher prevalence of extended β-lactamases producing *E. coli* (8).

Despite recent advances in developing antibacterial drugs, every infection is still a widespread, serious, and worldwide problem (9). This problem is accompanied by rapidly increasing multi-drug resistance (MDR) of microorganisms to antibacterial treatments (10). MDR can be mediated by complex mechanisms, such as overexpression of antibiotic-inactivating enzymes, lack of or an alteration in a target site, increased efflux, or lowered penetrability of the drug due to decreased permeability (11). The alarming increase in antibiotic-resistant bacteria highlights the urgent need for more effective drugs to combat bacterial infection (12). Although they do not possess significant antibacterial activity, they bind to β-lactamases and inactivate the β-lactamases, thereby protecting the antibiotics that are typical substrates for these enzymes (13). *Artemisia annua* L., an annual medicinal herb, can grow wild in China and Vietnam's temperate and high-altitude regions (11, 13). Artemisinin, one of the bioactive compounds with anti-malarial activity, has been successfully isolated from *A. annua* (14). Other than anti-malarial activity, it is used to alleviate high fever and treat jaundice (15). Artemisinin was an excellent antibacterial, antifungal, antileishmanial, and antitumor agent (16). Initially, artemisinin was isolated from the traditional Chinese herb *A. annua* L. (sweet wormwood), an active ingredient containing a sesquiterpene lactone (17). Artemisinin and its derivatives, such as artemisinin, dihydroartemisinin, and artesunate, have been widely used against malaria. Artemisinin and its derivatives have recently been found effective in treating viral infections and tumors (18, 19). Previously, we found that artemisinin and its derivatives could protect the sepsis model of mice from challenges with a heat-killed *E. coli* by reducing pro-inflammatory cytokine release and endotoxin level *via* suppressing the activation of toll-like receptor (TLR) 4/TLR9/nuclear factor-kB pathways (20, 21). The antibacterial properties of artemisinin have been tested on a wide range of bacteria, such as *E. coli, S. aureus, Pseudomonas aeruginosa*, and *Mycobacterium intracellulare* (22).

In brief, artemisinin and its derivatives (Dihydroartemisinin, Artesunate, and Artemisinic Acid) may become potential antibacterial candidates. Therefore, the enhancement of antibacterial activity of various β-lactam and fluoroquinolones antibiotics by Artemisinin and its Dihydroartemisinin against multiple drug resistant *E. coli* were objectives of this study.

## Materials and methods

### Chemical and antibiotics

Artemisinin, Dihydroartemisinin, Artesunate, and Artemisinic acid were obtained from the China Institute of food and drug control and dissolved following the instruction before use. All antibiotics (ciprofloxacin, imipenem, penicillin G, ampicillin, and oxacillin) were purchased from the North China Pharmaceutical Group Corp (Shijiazhuang, China) and Southwest Synthetic Pharmaceutical Co. Ltd (Chongqing, China). All antibiotics were dissolved and diluted according to Clinical and Laboratory Standards Institute (CLSI) guidelines (23). Antibiotics disks (10 μg) of all five antibiotics and strips of ciprofloxacin, imipenem (0.002–32 mg), penicillin G, ampicillin, and oxacillin (0.016–256 mg) were purchased from Liofilchem srl (Zona Industriale Italy). All standard strains of *E. coli* BNCC 186347, 125787, 125988, and 195617 were purchased from BeNe Culture Collection, Kunshan city, Jiangsu province, China.

### Bacterial inoculation

The bacterial suspension was prepared from overnight cultures by the direct colony method. Colonies were taken directly from the plate and suspended in 5 ml of sterile 0.85% saline. The turbidity of the initial suspension was adjusted compared with the 0.5 Mc Farland standard contains about 1 × 10^8^ colony forming units (CFUs)/ml. Ten-fold dilutions of the initial suspension were additionally prepared into sterile 0.85% saline to achieve 1 × 10^6^ CFU/ml (24).

### Preparation of antibiotic stock solution

Standard powder forms of penicillin, ampicillin, oxacillin, ciprofloxacin, and imipenem were stored at 4°C till usage. The stock solution of each antibiotic was prepared by weighing and consequently dissolving suitable amounts of the antibiotics, reaching a concentration of 1,024 μg/ml in Mueller-Hinton broth.

### Disk diffusion method

The Kirby-Bauer disk diffusion test performed the antimicrobial susceptibility test of the isolates (25). In brief, each test isolate was swabbed uniformly onto the surface of Mueller-Hinton agar plates (26). A drug-sensitive paper sheet containing (10 μg) penicillin, ampicillin, oxacillin, ciprofloxacin, and imipenem was pasted on the agar plate inoculated with the bacteria to be tested. Following incubation, the inhibition zones, in millimeters, were measured in duplicate and scored as sensitive, intermediate, and resistant categories by the critical breakpoints recommended by the Clinical and Laboratory Standards Institute (23).

### Determination of the minimum inhibitory concentration

Minimum inhibitory concentration (MIC) values of antibiotics were determined by the microdilution method following the recommendations of Papich (27). Stock solutions of antibiotics were prepared and added to the bottom of a 96-well microtiter plate (Nunc Inc., Roskilde, Denmark). The first well of the 96-well plate was filled with 100 μl of the stock solution, which was then serially diluted. Each well received 100 μl of an overnight *E. coli* culture at a final concentration of 5 × 10^5^ CFU/ml. The microtiter plates were incubated at 35°C for 24 h, and the MIC was determined as the lowest concentration of antibiotics showing no visible bacterial growth (28).

### Synergy testing

The synergistic effect of the antibiotic combinations was detected using a checkerboard dilution assay (29). The initial concentration of each drug was twofold greater than the MIC concentration. In a screw cap test tube, 0.25 ml of broth of each two drugs to be tested was added to 0.5 ml of broth containing a suspension of the organism to be tested to reach the final volume of 1 ml. The inoculum of the bacterial suspension (in 0.5 ml of broth) was 2 × 10^5^ CFU to produce a final inoculum of 1 × 10^5^ CFU per ml after adding an equal volume of the antimicrobial solutions. Each test was composed of 36 tubes set horizontally and vertically. Six rows in one direction contained twofold serial dilutions of antibiotic, and six rows in the other direction contained twofold serial dilutions of the drug; two additional rows had twofold serial dilutions of antibiotic and drug alone. The tubes were incubated at 37°C for 24 and 48 h, the tubes were read as those showing turbidity (+) and those showing no turbidity (–). Also, swabs from each tube were streaked on blood agar plates. These plates were seen as those showing growth and those showing no growth. A fractional inhibitory concentration index was used to interpret the results. The FIC of each agent was calculated by dividing the MIC of the drug in combination with the MIC of the drug alone as mentioned in the equations below.


$$\begin{array}{l} FIC\;of\;Drug\;A=\frac{MIC\;of\;drug\;A\;in\;presence\;of\;drug\;B}{MICof\;drug\;A\;\left(Alone\right)}\\ FIC\;of\;Drug\;B=\frac{MIC\;of\;drug\;B\;in\;presence\;of\;drug\;A}{MICof\;drug\;B\;\left(Alone\right)}\\ \;FIC\;Index=FIC\;of\;drug\;A+FIC\;of\;drug\;B \end{array}$$

The sum of both FICs (∑FIC = FIC of antibiotic + FIC of Drug) in each well was used to categorize the combined activity of antimicrobial agents at the given concentrations as synergistic (0 < FICI ≤ 0.5), additive (0.5 < FICI ≤ 1), indifferent (1 < FICI ≤ 4), and antagonistic (∑FICI ≥4) [33].

### Unit change/percent change in the efficacy of antibiotics

Unit increase or decrease in MIC of antibiotics used in combination with the drug compared to the antibiotic used alone was calculated (formula given below) to find differences among different interactions at the level of 1 μg/ml of antibiotic used alone. To develop a more general understanding, the percentage change in MIC of antibiotics used in combination compared to that used alone was calculated (formula given below).


$$\begin{array}{l} \text{A unit change in MIC}=\;\left(MIC\;in\;combination-MIC\;alone\right)/\\ \;MIC\;alone \end{array}$$

$$\begin{array}{l} \text{Percentage change in MIC of antibiotic}\\ =\left(\left(\text{MIC in combination}-\text{MIC alone}\right)/\text{MIC alone}\right)\;\times 100 \end{array}$$

### Killing kinetics

Time–kill assays on *E. coli* were performed using artemisinin, dihydroartemisinin, artesunate, and artemisinic acid at 0.5 × , 1 × , and 2 × the MIC. After 24 h and 48 h of incubation, the number of CFU was assessed by serial dilution. The rate and extent of killing were expressed as viable count (log_10_ CFU/ml) against time (27).

### First significant reduction in bacterial count (μg/ml)

All drug-antibiotic interactions were further analyzed for their quick response. The bacterial count was assessed by their first significant reduction in MIC among different time intervals.

### Comparison of between drugs used in combination with antibiotics

To highlight which drug showed a higher effect in combination with antibiotics, the comparison between ART and DHA when used with each antibiotic and each time interval was analyzed. Similarly, AS and AA was compared by comparing MIC of drug-antibiotic combinations.

### Statistical analysis

SPSS version 22 for Windows was used to analyze the data. A *t*-test was used to find statistical differences between the experiments, such as the difference between using a combination of antibiotics and just one antibiotic. One-way ANOVA was also used to compare how these treatments affected the fold change of MIC values. It was suspected that a *p* ≤ 0.05 value was statistically significant.

## Results

### Antibacterial response

The study found a significant difference in minimum inhibitory concentrations (MIC) among tested drugs in that DHA and AA differed significantly (*p* < 0.05) between each other and with ART and AS. However, ART and AS showed a non-significant difference (*p* > 0.05) in MIC. ART showed the highest MIC, while DHA expressed a minimum of all four drugs against *E. coli*, exhibiting that the latter showed the highest antibacterial potential among the slots (Supplementary Figure S4).

Antibiotic resistance was confirmed by all three phenotypic methods commonly used against bacteria (Table 1, Supplementary Figure S1). In E-test, ciprofloxacin stood highly productive because it showed a minor MIC (0.017125 ± 0.0022 μg/ml), followed by imipenem, ampicillin, penicillin, and oxacillin. The latter showed a very high MIC (256 μg/ml). Similar findings were noted when the disc diffusion test and broth microdilution methods were applied. MIC values were, however, higher in the case of the broth microdilution method compared to the E-strip test.

**Table 1:** Confirmation of antibiotic resistance profile of *E. coli* against different antibiotics using different tests.

**Antibiotic**	**E-test**	**ZOIs**	**Broth microdilution**
**names**	**(μg/ml)**	**(mm)**	**method MICs (μg/ml)**
Penicillin	172 ± 88.63	1.245 ± 1.376	215 ± 251.147
Ampicillin	71.125 ± 106.882	14.545 ± 8.97	170.94 ± 271.26
Oxacillin	256 ± 0	0 ± 0	360 ± 174.36
Ciprofloxacin	0.017125 ± 0.0022	37.12 ± 1.596	0.0196 ± 0.012
Imipenem	0.239 ± 0.09262	29.035 ± 1.317	0.625 ± 0.383

### Synergism of ART and DHA with antibiotics

The study showed none of the antagonistic responses of ART with all the antibiotics. A similar response was noted in the case of DHA with antibiotics (Table 2, Supplementary Figure S2). The latter showed no interaction as indifferent. In contrast, the former showed 25% of isolates falling in the indifferent category when ART was combined with ciprofloxacin, ampicillin, and oxacillin, while with imipenem, 50% fell in the stated category. Partial synergism was noted highest response compared to all other interactions when ART and DHA were combined with antibiotics.

**Table 2:** Percentage of isolates with different synergy interactions of ART and DHA with antibiotics.

**Drug name**	**Antibiotic name/type**	**Type of synergy interactions (%)**			
	**of drug interactions**	
		**Synergistic**	**Additive**	**Indifferent**	**Antagonistic**
**ART**.	Ciprofloxacin	25	50	25	0
	Imipenem	0	50	50	0
	Ampicillin	0	75	25	0
	Oxacillin	0	75	25	0
	Penicillin G	50	50	0	0
DHA.	Ciprofloxacin	50	50	0	0
	Imipenem	0	100	0	0
	Ampicillin	0	100	0	0
	Oxacillin	25	75	0	0
	Penicillin G	50	50	0	0

In combination with antibiotics, AS and AA showed higher percentages of isolates falling into the sensitive category than ART and DHA (Tables 2, 3). All the *E. coli* isolates showed partial and complete synergism when AA was used in combination with antibiotics against (Table 3). Penicillin G, ampicillin, and oxacillin in combination with AA showed 100, 75, and 50% of isolates falling in the complete sensitive category, while imipenem and ciprofloxacin showed 75 and 50% of isolates falling into partial sensitive drug interaction. On the other hand, artesunate, in combination with imipenem, oxacillin, and ciprofloxacin, showed 75 75, and 50% of isolates showed partial synergism.

**Table 3:** Percentage of isolates with different synergy interactions of AA and AS with antibiotics.

**Drug**	**Antibiotic**	**Type of synergy**			
**name**	**name**	**interactions (%)**			
		**Synergistic**	**Additive**	**Indifferent**	**Antagonistic**
**AA**	Ciprofloxacin	50	50	0	0
	Imipenem	25	75	0	0
	Ampicillin	75	25	0	0
	Oxacillin	50	50	0	0
	Penicillin G	100	0	0	0
AS	Ciprofloxacin	0	50	50	0
	Imipenem	0	75	25	0
	Ampicillin	25	50	25	0
	Oxacillin	0	75	25	0
	Penicillin G	50	25	25	0

### Change in MIC of antibiotics in combination with ART and DHA

The analysis of unit changes in MIC of antibiotics in combination with drugs (ART & DHA) compared to the antibiotics used alone showed the highest decrease in the case of oxacillin with ART and ampicillin with DHA (Table 4, Supplementary Figure S3). The comparison of the unit decreases for ART and antibiotics was found uniform as there was a non-significant difference among antibiotics in combination with ART. The same was noted among antibiotics combined with DHA.

**Table 4:** Comparative unit (means ± SD) change in minimum inhibitory concentration (μg/ml) of antibiotics used in combination with ART and DHA.

**Antibiotics**	**ART**	**DHA**
Penicillin G	−0.888 ± 0.0729^a^	−0.875 ± 0.068^a^
Oxacillin	−0.925 ± 0.9225^a^	−0.762 ± 0.728^a^
Ampicillin	−0.631 ± 0.758^a^	−0.844 ± 0.825^a^
Imipenem	−0.7624 ± 0.727^a^	−0.812 ± 0.787^a^
Ciprofloxacin	−0.755 ± 0.718^a^	−0.870 ± 0.892^a^

Different superscripts within a column indicate significant differences (p < 0.05). The formula “Unit change in MIC = (MIC in combination-MIC alone)/MIC alone.”

### Change in MIC of antibiotics in combination with AS and AA

A comparison of a unit change in MIC of antibiotics in combination with AA and AS showed a non-significant difference (*p* > 0.05) with each other indicating a smooth response by drug interaction assay (Table 5). A more remarkable change in MIC was noted in the case of ampicillin (−0.912 ± 0.908 μg/ml) in combination with AA, followed by ciprofloxacin (−0.901 ± 0.893 μg/ml) combination while in the case of AS; there was a more significant change in penicillin G (−0.9 ± 0.0306 μg/ml). The fact indicates that 0.912 ± 0.908 μg/ml of ampicillin antibiotic was reduced in combination with AA compared to 1 μg/ml of it used alone. The same goes for others in this trial, where a significant reduction in MIC was observed. Expressing percentage decrease in MIC was found more in penicillin G and oxacillin when combined with ART than DHA (Supplementary Figure S5). Ampicillin, imipenem, and ciprofloxacin showed a more significant reduction in MIC when combined with DHA. However, penicillin G showed a more substantial decrease in MIC when combined with AS, while all other antibiotics showed more MIC reduction when combined with AA (Supplementary Figure S6).

**Table 5:** Comparative unit (means ± SD) change in minimum inhibitory concentration (μg/ml) of antibiotics used in combination with AA and AS.

**Antibiotics**	**AA**	**AS**
Penicillin G	−0.8375 ± 0.075^a^	−0.9 ± 0.0306^a^
Oxacillin	−0.8375 ± 0.816^a^	−0.8 ± 0.81^a^
Ampicillin	−0.912 ± 0.908^a^	−0.894 ± 0.885^a^
Imipenem	−0.800 ± 0.810^a^	−0.850 ± 0.833^a^
Ciprofloxacin	−0.901 ± 0.893^a^	−0.851 ± 0.834^a^

Different superscripts within a column indicate significant differences (p < 0.05). Formula “Unit decrease = (MIC in combination-MIC alone)/MIC alone.”

### Time kill assay and first reduction in bacterial count (CFU/ml)

The first reduction in MIC was described to find earlier efficacy of antibiotics with drug combinations against *E. coli* to provide a baseline for adequate time intervals for dose regimens. Time kill assay showed zero bacterial counts after 24 h of incubation of *E. coli* against ciprofloxacin in combination with AS and penicillin G in combination with AS and AA. In contrast, all other combinations of antibiotics with drugs showed some bacterial count (CFU/ml) (Table 6). The study showed a significant bacterial count reduction started at the 4th hour of incubation in case ciprofloxacin combined with AS and penicillin G in combination with AA. At the 6th hour of incubation, the first significant reduction of MIC was noted in the case of ampicillin and imipenem were used in combination with Similarly, ciprofloxacin with AA, ampicillin with AS, oxacillin with AS, and penicillin G with AS showed the first reduction in their MICs at 8th hour of incubation. It was noteworthy that oxacillin in combination with AA could show the first reduction in MIC after 24 h of incubation.

**Table 6:** Comparison of time kills assay (bacterial count, CFU/ml) under the effect of AS and AA.

**Name of antibiotic**	**Drug name**	**0 h**	**2 h**	**4 h**	**6 h**	**8 h**	**24 h**
Ciprofloxacin	AS	297.0 ± 156.42^a^	166.0 ± 174.02^a^	72.00 ± 82.126^b^	40.25 ± 42.161^c^	5.75 ± 11.50^d^	0.00 ± 0.00^e^
	AA	447.00 ± 254.649^a^	264.25 ± 303.983^a^	185.25 ± 218.007^a^	66.75 ± 82.062^a^	24.50 ± 32.265^b^	3.25 ± 6.500^c^
Imipenem	AS	292.75 ± 256.860^a^	151.25 ± 251.900^a^	123.75 ± 208.823^a^	102.25 ± 196.581^a^	69.50 ± 139.000^a^	39.00 ± 78.00^a^
	AA	596.50 ± 141.6^a^	496.25 ± 144.7^a^	400.00 ± 163.1^a^	248.25 ± 168.8^b^	54.00 ± 68.13^c^	1.00 ± 2.000^d^
Ampicillin	AS	335.25 ± 216.5^a^	280.00 ± 189.2^a^	206.50 ± 152.5^a^	112.50 ± 88.98^a^	28.50 ± 37.89^b^	10.25 ± 20.5^c^
	AA	441.00 ± 186.790^a^	300.00 ± 213.815^a^	235.00 ± 222.577^a^	90.251 ± 08.014^b^	21.50 ± 36.638^c^	1.00 ± 2.000^d^
Oxacillin	AS	385.25 ± 226.553^a^	271.25 ± 196.315^a^	218.25 ± 155.127^a^	136.75 ± 140.555^a^	22.50 ± 26.032^b^	0.50 ± 1.000^c^
	AA	374.25 ± 187.416^a^	269.75 ± 199.796^a^	169.00 ± 184.808^a^	123.25 ± 163.700^a^	68.50 ± 99.915^a^	24.25 ± 48.500^b^
Penicillin G	AS	290.75 ± 198.028^a^	143.75 ± 131.677^a^	91.00 ± 140.490^a^	62.50 ± 100.125^a^	6.00 ± 12.000^b^	.00 ± .00^c^
	AA	357.00 ± 199.371^a^	177.25 ± 107.608^a^	128.75 ± 85.745^b^	38.25 ± 46.843^c^	17.00 ± 24.042^d^	0.00 ± 0.00^e^

Different superscripts within a row indicate a significant difference (p < 0.05).

ART and DHA showed better antibiotic interaction than AS and AA against *E. coli*. There was no bacterial count (CFU/ml) after the 8^th^ and 24^th^ hour of incubation when ciprofloxacin was used with ART, while other zero CFU/ml were noted after the 24^th^ hour of all different drug combinations except for imipenem with ART and DHA; and penicillin G with DHA (Table 7). The first reduction in bacterial count (CFU/ml) was started at 2 h of incubation in the case of ciprofloxacin with ART. In contrast, at the 4th-hour incubation, this response was noted in the case of penicillin in combination with ART. Only ampicillin with ART showed a first significant reduction in MIC at the 6th hour of incubation.

**Table 7:** Comparison of the time-kill assay (bacterial count) under the effect of ART and DHA.

**Name of antibiotic**		**0 h**	**2 h**	**4 h**	**6 h**	**8 h**	**24 h**
Ciprofloxacin	ART	190.25 ± 125.128^a^	13.00 ± 16.269^b^	4.50 ± 8.347^c^	2.50 ± 5.00^d^	0.00 ± 0.00^e^	0.00 ± 0.00^e^
	DHA	326.75 ± 303.418^a^	239.00 ± 278.713^a^	195.00 ± 252.521^a^	113.00 ± 192.897^a^	8.25 ± 16.500^a^	0.00 ± 0.00^b^
Imipenem	ART	287.50 ± 239.511^a^	174.75 ± 246.384^a^	126.25 ± 210.270^a^	107.75 ± 193.498^a^	4.50 ± 9.000^a^	2.00 ± 4.00^a^
	DHA	503.75 ± 187.41^a^	381.00 ± 177.40^a^	253.25 ± 212.734^a^	193.25 ± 182.872^a^	44.00 ± 48.833^b^	22.50 ± 35.707^c^
Ampicillin	ART	308.50 ± 168.761^a^	193.75 ± 189.804^a^	111.00 ± 121.403^a^	47.50 ± 75.372^b^	11.25 ± 16.029^c^	0.00 ± 0.00^d^
	DHA	357.00 ± 211.781^a^	286.25 ± 232.213^a^	169.00 ± 177.281^a^	108.50 ± 119.551^a^	2.75 ± 4.272^b^	0.00 ± 0.00^c^
Oxacillin	ART	286.75 ± 144.657^a^	183.50 ± 173.221^a^	144.00 ± 159.689^a^	69.50 ± 111.683^a^	14.25 ± 20.271^b^	0.00 ± 0.00^c^
	DHA	386.75 ± 238.315^a^	288.75 ± 262.500^a^	183.50 ± 208.058^a^	116.25 ± 127.369^a^	34.50 ± 54.751^b^	0.00 ± 0.00^c^
Penicillin G	ART	300.00 ± 161.107^a^	123.75 ± 83.731^a^	60.50 ± 103.989^b^	56.25 ± 96.472^c^	8.50 ± 10.116^d^	0.00 ± 0.00^e^
	DHA	374.50 ± 262.062^a^	265.25 ± 258.965^a^	125.50 ± 204.386^a^	91.75 ± 156.585^a^	16.25 ± 18.768^a^	5.50 ± 11.00^a^

In contrast, at the 8th hour of incubation, imipenem with DHA, ampicillin with DHA, oxacillin with ART, and DHA oxacillin with DHA did not significantly reduce bacterial count (CFU/ml) throughout the time-kill assay. It was also noted that there were higher standard deviations and some of the combinations, which reflected significant variations of response existing with strains of *E. coli*.

### Comparison between drugs in a combination of each antibiotic at different time intervals

**Figure 1:**
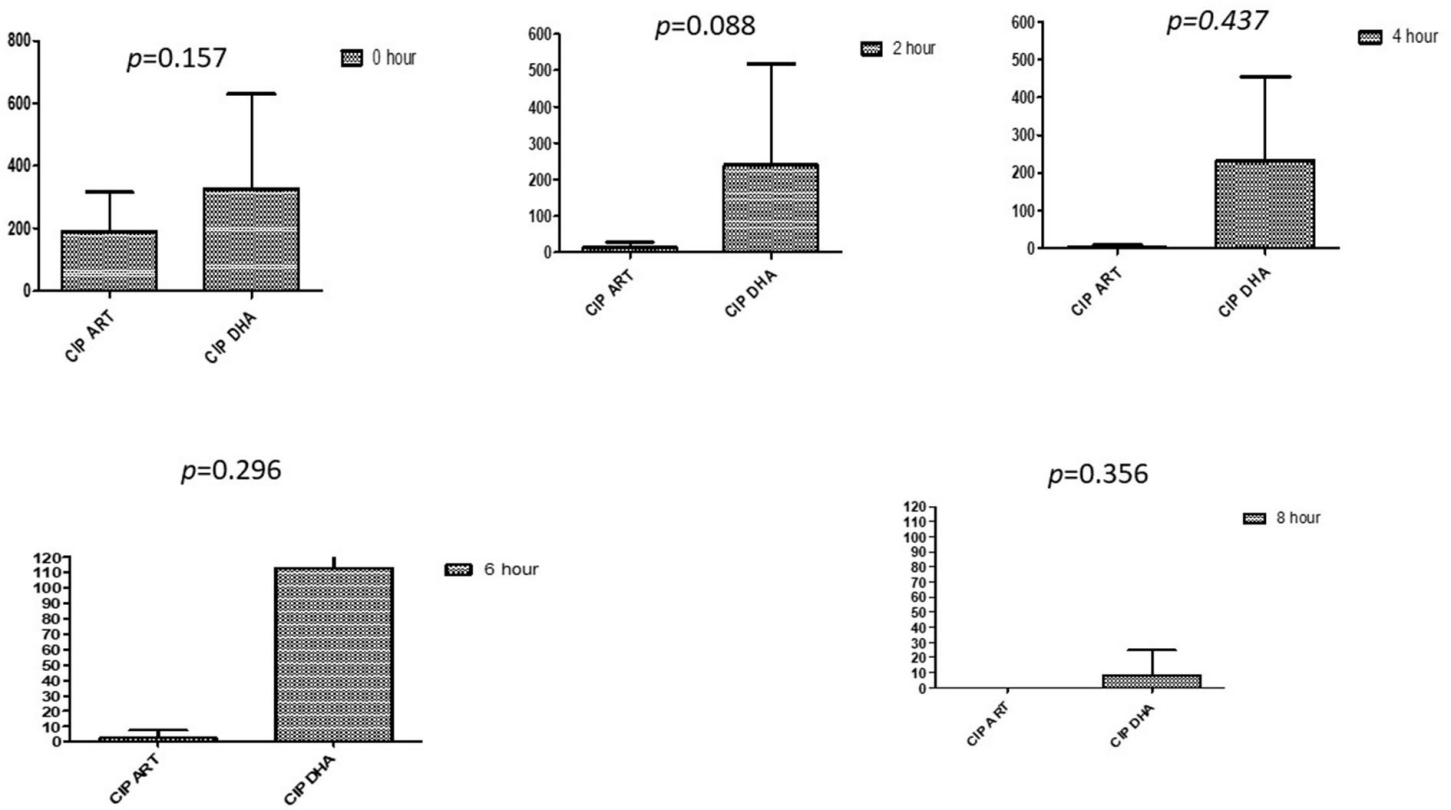
Compare ciprofloxacin's minimum inhibitory concentrations (μg/mL) when combined with ART and DHA on E. coli. CIP ART, Ciprofloxacin combined with ART; CIP DHA, Ciprofloxacin combined with DHA; p < 0.05 indicate a significant difference among MICs of antibiotic combined ART and DHA.

**Figure 2:**
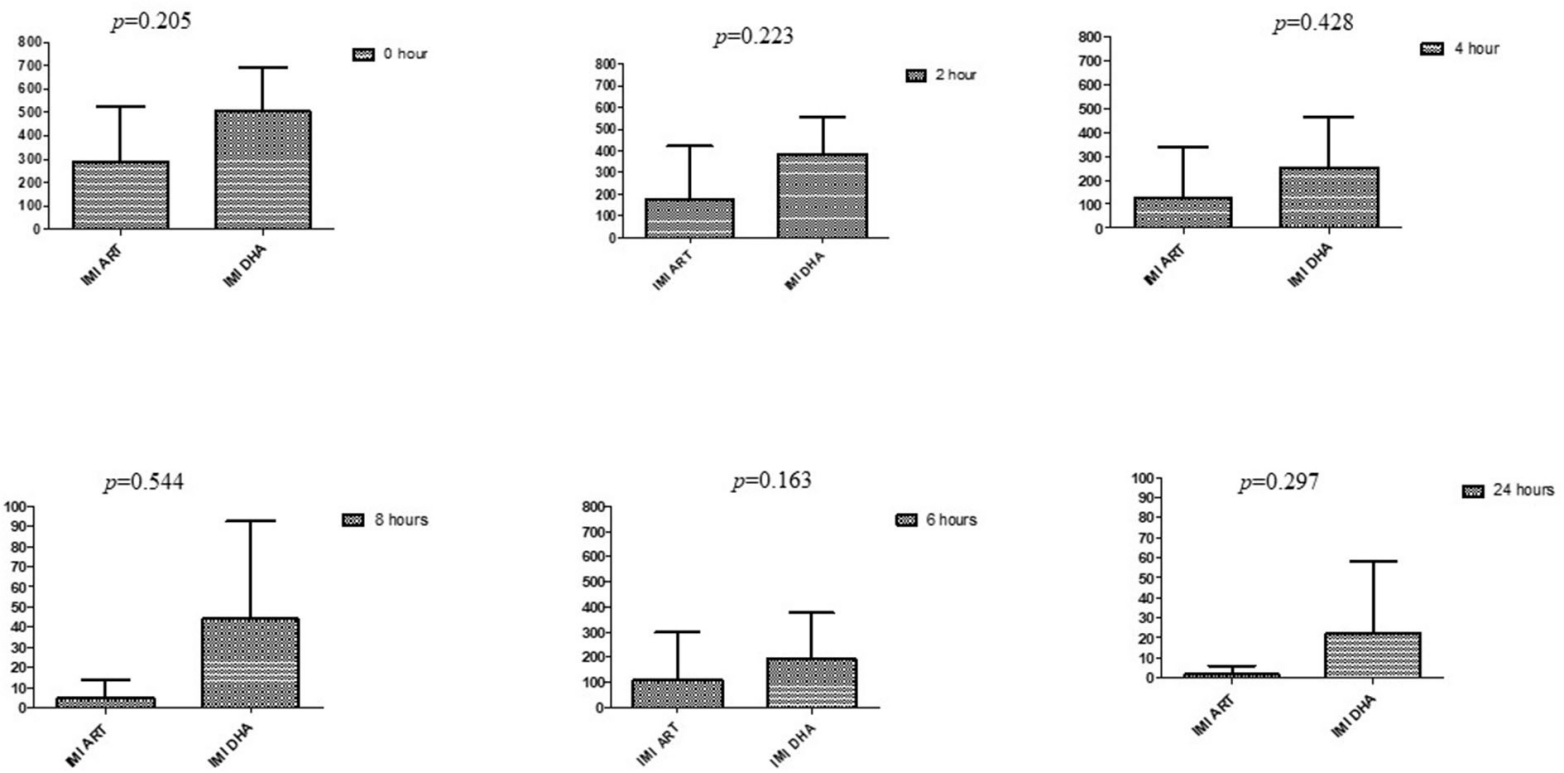
Comparison of imipenem's minimum inhibitory concentrations (μg/mL) when combined with ART and DHA on E. coli. IMI ART, Imipenem combined with ART; IMI DHA, Imipenem combined with DHA; p < 0.05 indicate a significant difference among MICs of antibiotic combined with ART and DHA.

**Figure 3:**
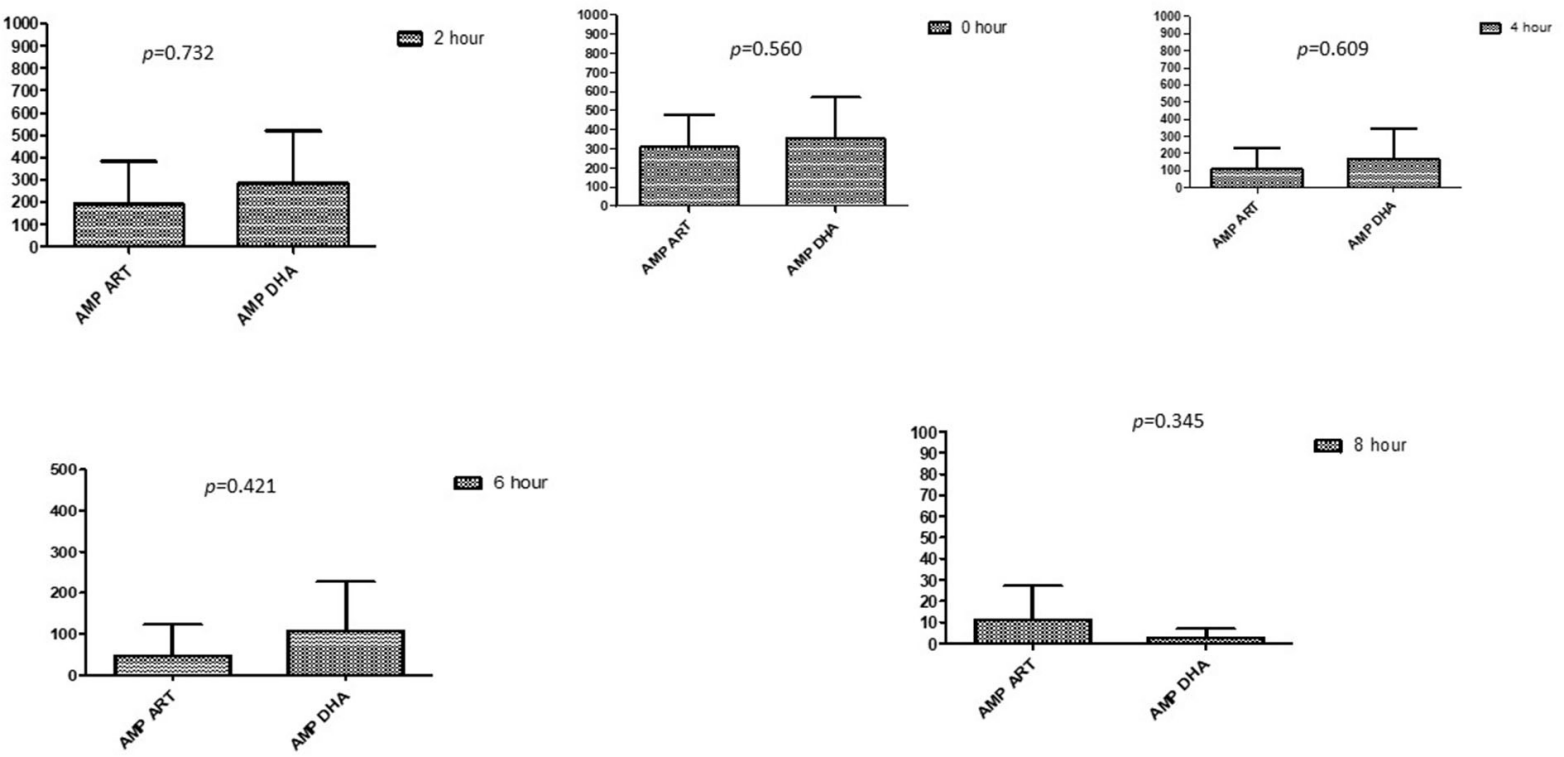
Comparison of ampicillin's minimum inhibitory concentrations (μg/mL) when combined with ART and DHA on E. coli. AMP ART, Ampicillin combined with ART; AMP DHA, Ampicillin combined with DHA; p < 0.05 indicate a significant difference among MICs of antibiotic combined ART and DHA.

**Figure 4:**
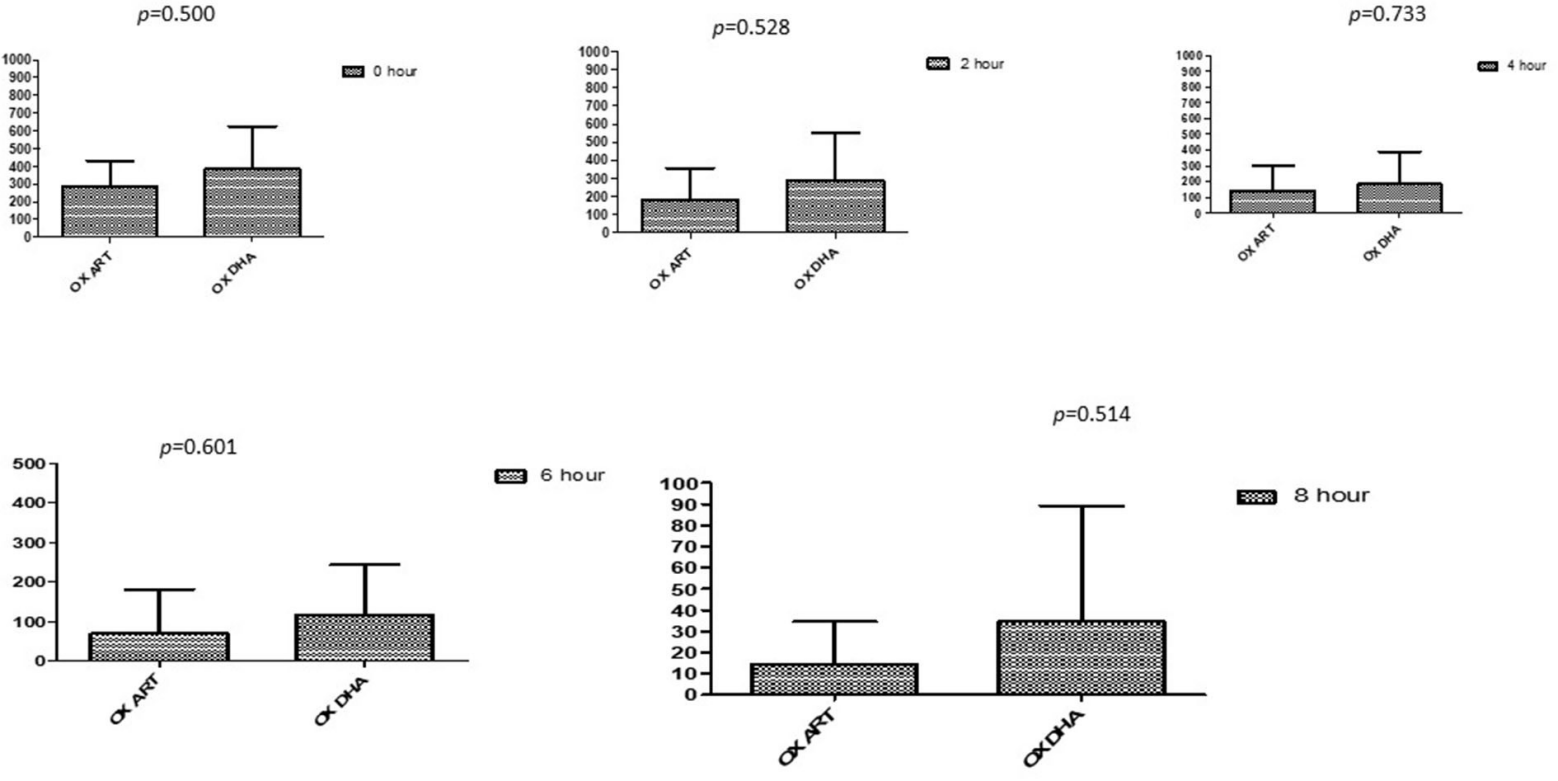
Comparison of Oxacillin's minimum inhibitory concentrations (μg/mL) when combined with ART and DHA on E. coli. OX ART, Oxacillin combined with ART; OX DHA, Oxacillin combined with DHA; p < 0.05 indicate a significant difference among MICs of antibiotic combined ART and DHA.

**Figure 5:**
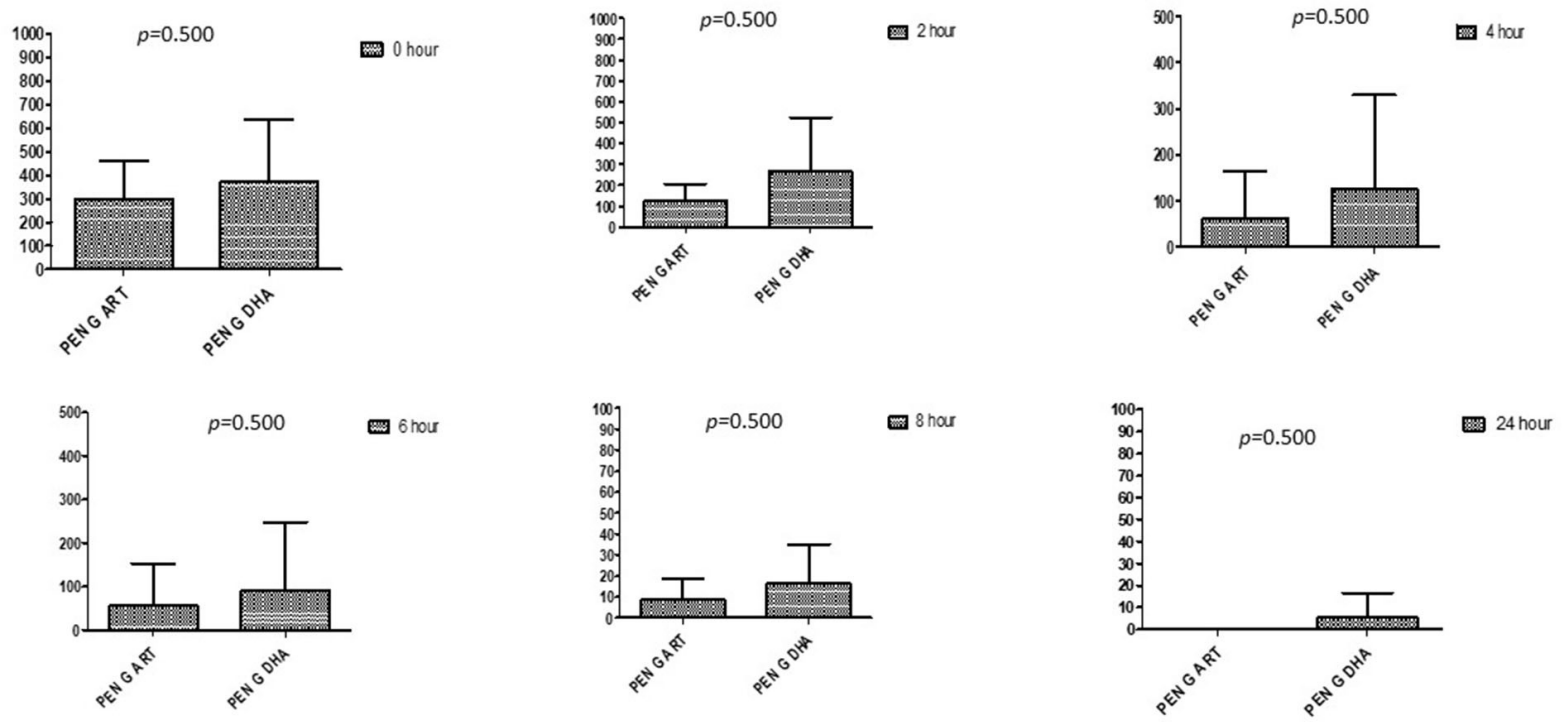
Comparison of minimum inhibitory concentrations (μg/mL) of Penicillin G when combined with ART and DHA on E. coli. PENG ART, Penicillin G combined with ART; PEN G-DHA, Penicillin G combined with DHA; p < 0.05 indicate a significant difference among MICs of antibiotic combined with ART and DHA.

**Figure 6:**
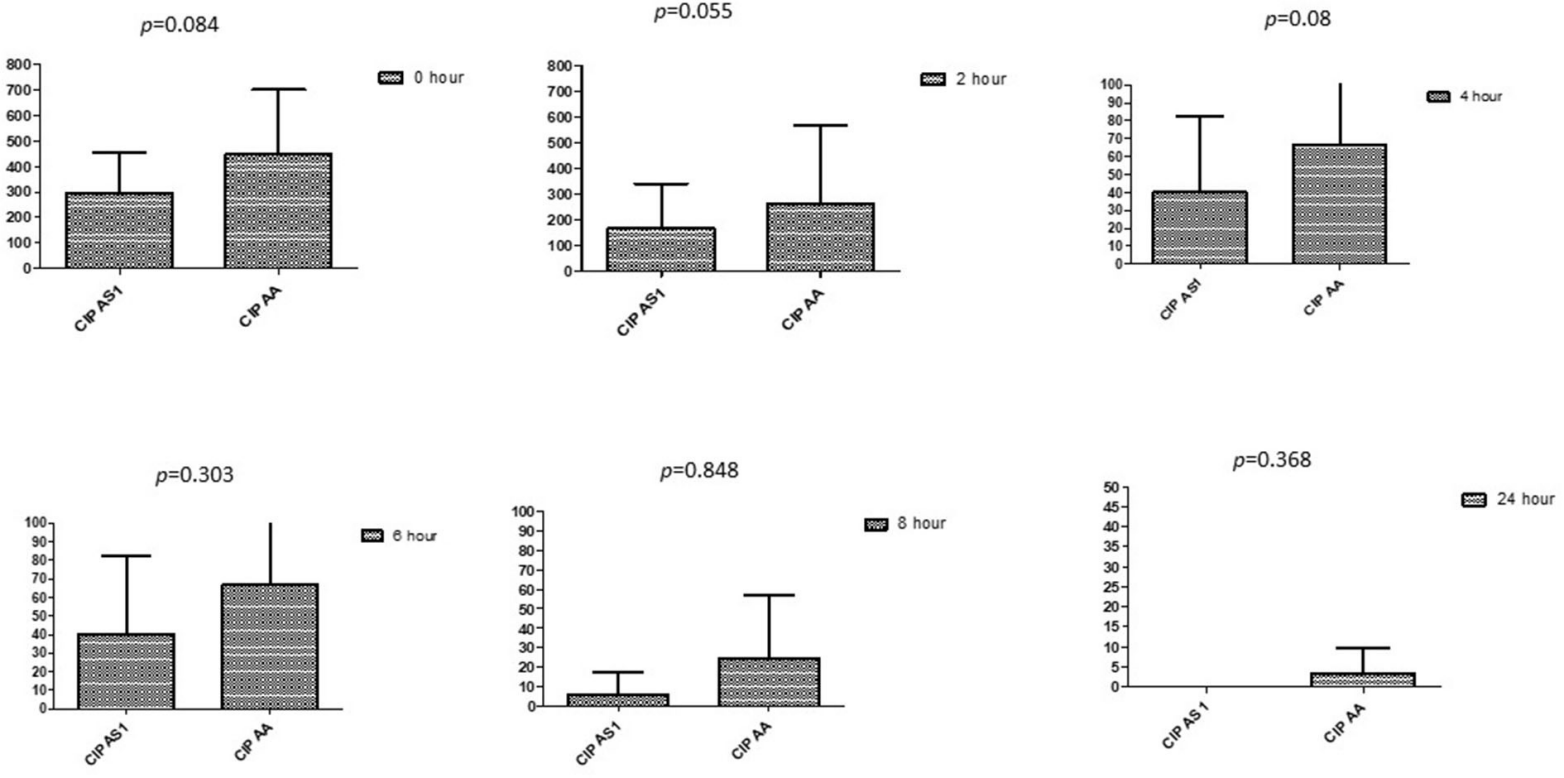
Comparison of ciprofloxacin's minimum inhibitory concentrations (μg/mL) when combined with AA and AS on E. coli. CIP AS, Ciprofloxacin combined with AS; CIP AA, Ciprofloxacin combined with AA; p < 0.05 indicate a significant difference among MICs of antibiotic combined AS1 and AA.

**Figure 7:**
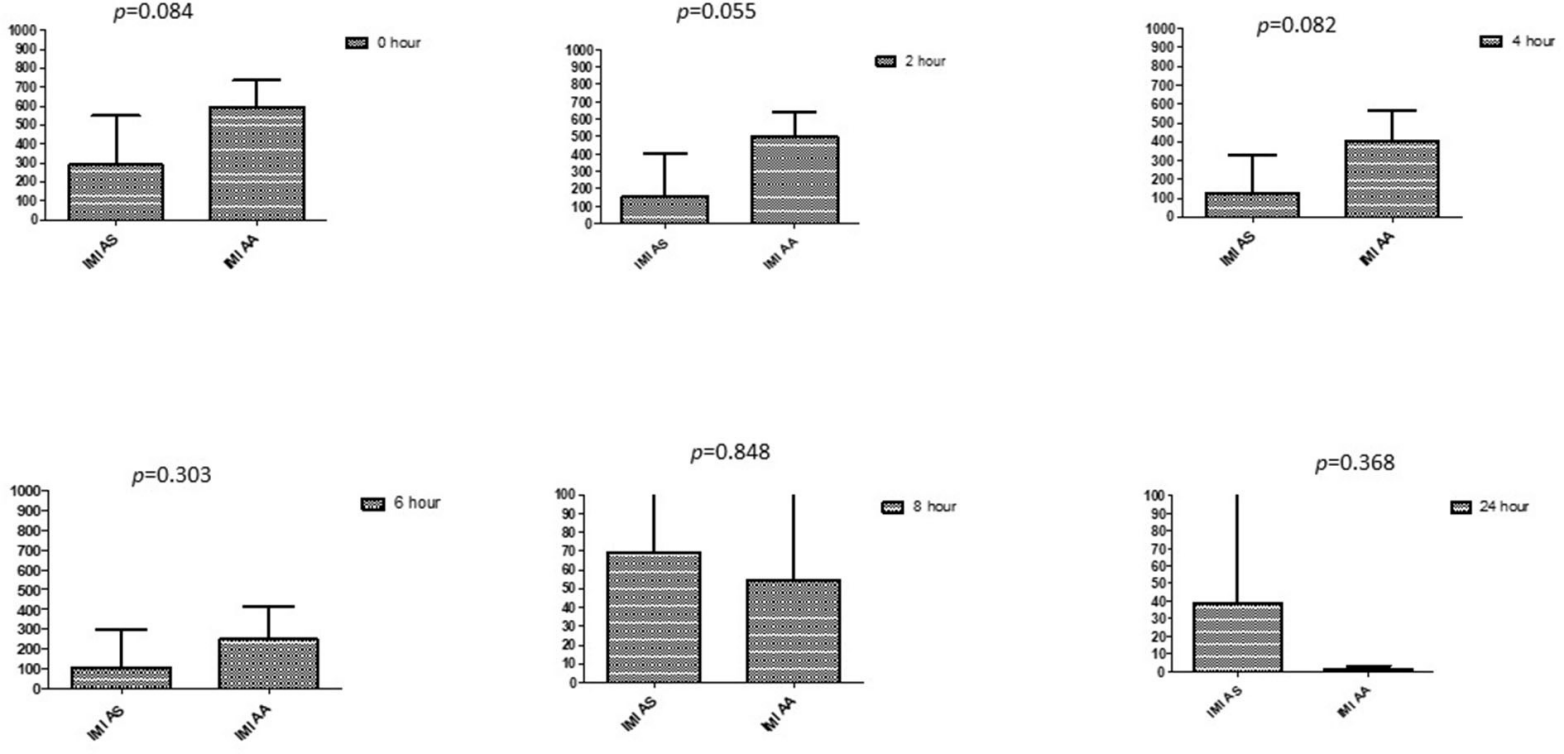
Comparison of imipenem's minimum inhibitory concentrations (μg/mL) when combined with AA and AS on E. coli. IMI-AS, Imipenem combined with AS; IMI-AA, Imipenem combined with AA; p < 0.05 indicate a significant difference among MICs of antibiotic combined with AS and AA.

**Figure 8:**
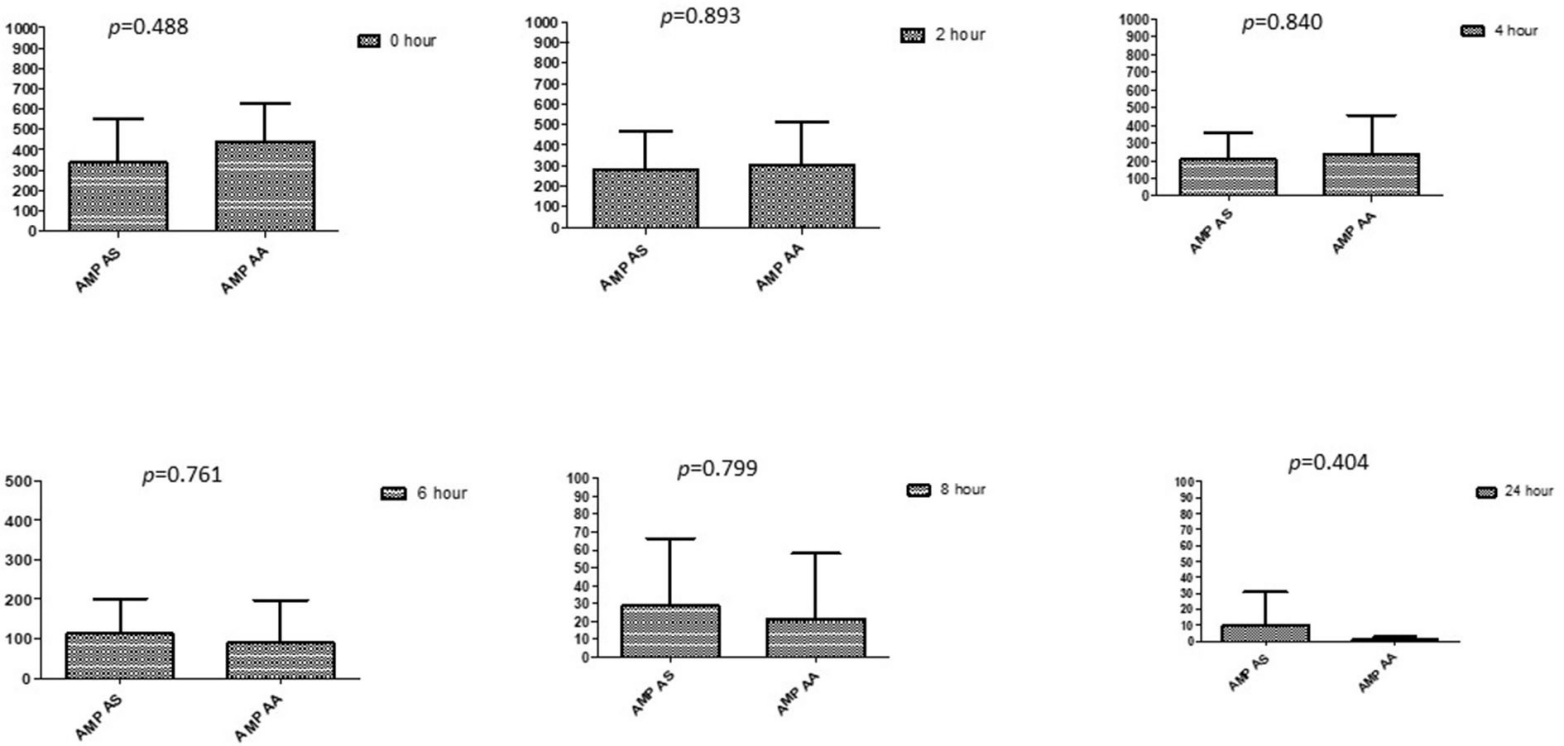
Comparison of ampicillin's minimum inhibitory concentrations (μg/mL) when combined with AA and AS on E. coli. AMP-AS, Ampicillin combined with AS; AMP-AA, Ampicillin combined with AA; p < 0.05 indicate a significant difference among MICs of antibiotic combined with AS and AA.

**Figure 9:**
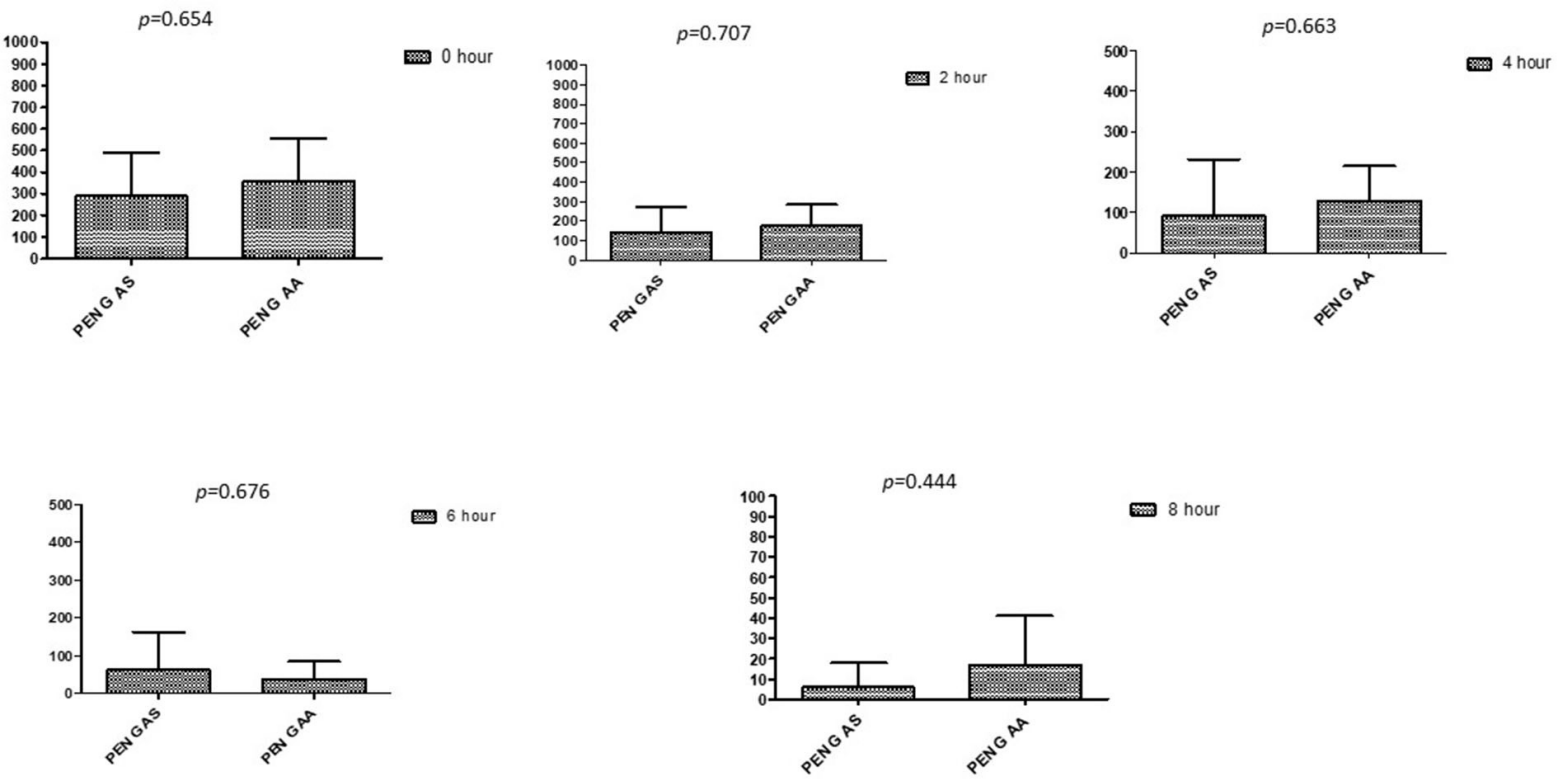
Comparison of minimum inhibitory concentrations (μg/mL) of Penicillin G when combined with AA and AS on E. coli. PEN G-AS, Penicillin G combined with AS; PEN G-AA, Penicillin G combined with AA; p < 0.05 indicate a significant difference among MICs of antibiotic combined with AS and AA.

This section described the difference between ART and DHA (*t*-test) when combined with antibiotics at 2, 4, 6, 8, and 24^th^ hour of incubation. Similarly, AA and AS were compared with each antibiotic to find a better efficacious drug (Figures 1–9). The comparison between ART and DHA in combination with ciprofloxacin showed a non-significant (*p* > 0.05) difference at 2, 4, 6, and 8th hour of incubation (Figure 1). A similar response of comparison of ART and DHA with each imipenem, ampicillin, oxacillin, and penicillin G was noted at each time interval (Figures 6–9). A similar response was noticed when AA and AS were compared with each antibiotic at every incubation period (Figures 6–9). However, a comparison of AS and AA in combination with ciprofloxacin showed *p* = 0.055 and *p* = 0.088 at 2 and 4^th^ hour, respectively, of incubation. Similar *p*-values (*p* = 0.084, *p* = 0.055, and *p* = 0.082) were found at 0, 2, and 4 h of incubation when AA and AS in combination with imipenem were compared. Overall, all drugs presented continued support in reducing the antibiotic quantity to combat *E. coli*.

## Discussion

The anti-malarial drugs artemisinin and its derivatives (artesunate, dihydroartemisinin, artemether, and arteether) have been clinically used to treat malaria (18, 19, 30). Artesunate was also revealed to increase the susceptibility of various β-lactam antibiotics against *E. coli* by increasing antibiotics accumulation *via* inhibiting the efflux pump system, AcrAB-TolC, an essential and significant multi-drug efflux pump system within *E. coli* (31, 32). Results showed that oxacillin had the greatest MIC, while ciprofloxacin had the lowest MIC, supported by those reported by CLSI (23). The antibacterial activity of drugs at higher concentrations in the current study was in line with the previous research (33). However, in contradiction to the current study, Wu et al. (34) did not find the antibacterial activity of DHA and ART. Variation in the activity of DHA and AA from each other was in line with the findings of Wang et al. (35). Resistance to antibiotics using all three phenotypic tests was in line with Berge et al. (36). However, the broth microdilution method identified a higher MIC than the *E*-strip test (23).

No antagonistic effect of ART and DHA with all antibiotics was also noted (37). DHA has not shown indifferent interaction but synergistic and additive effects with antibiotics, previously described as an antitumor anti-malarial agent by Wu et al. (37). The additive effect of AS with imipenem, oxacillin, and ciprofloxacin was once also identified (38). Huang et al. (39) has also observed the property of artemisinin and its derivatives, as found in the current study. A comparison of antibiotic MIC changes with AA and AS revealed no significant difference (*p* > 0.05), confirming a smooth response by drug interaction test. To the best of our knowledge, these responses were seen by Li et al. (20) while using AS, but for AA, research was not found so far. The enhancement in antibiotic property of AA in the current study contradicted the previous studies that noted no or negligible antibiotic activity. Wu et al. (34) observed a reduction in penicillin G's MIC by AS than by AA while using ART and DHA with different antibiotics. Jingsu Meng also observed these values using ART, AS, and DHA (40). However, no AA higher standard deviations study for some combinations reflected substantial variability in *E. coli* reactivity. All drugs tested in the (41) study showed lower *E. coli* burdens. In our study, all four drugs, including ART, AS, DHA, and AA, have shown similar effects in combination with different antibiotics. It might be due to the effect described by Baucheron S (42). In addition to drug accumulation within the bacterial cell, it might also be possible to inhibit some drug transportation channels, Odds saw this effect when using Artemisinin (9). ART and AS were previously studied, but minimal information is available in combination with fluoroquinolones. No drug has shown an antagonistic effect when used with antibiotics, indicating that all drugs play an equally important role in lowering antibiotic resistance.

## Conclusion

The current study presented the enhanced activity of antibiotics in combination with artemisinin and its derivatives. Time-kill assay showed significant efficacy of synergistic combinations at earlier hours of incubation, indicating their potential to use in outbreaks. The study thus proposes that apart from developing new antibiotics, existing ones with enhancing monomers like artemisinin and its derivative be employed. Further studies are required to document efficacy and safety parameters in lab animals and at the farm level.

## Data availability statement

The original contributions presented in the study are included in the article/Supplementary material, further inquiries can be directed to the corresponding author.

## Author contributions

Conceptualization and methodology: SU, LW, JL, and WG. Validation: JL, LW, and WG. Formal analysis: SU, LW, AA, AM, and MS. Investigation: SU and LW. Data curation: SU, AA, and LW. Writing—original draft preparation and visualization: SU. Writing—review and editing: AA, SU, AM, MG, AI, SA, and KC. Supervision and funding acquisition: JL. Project administration: JL and LW. All authors contributed to the article and approved the submitted version.

## Funding

This work was supported by the Agricultural Science and Technology Innovation Program of the Chinese Academy of Agriculture Science (Grant No. CAAS-ASTIP-2014-LIHPS-04), Special Fund of the Chinese Central Government for Basic Scientific Research Operations in Commonweal Research Institute (Grant No. 1610322020005), Natural Science Foundation of Gansu Province (Grant No. 18JR3RA398), Science and Technology Program of Gansu (Grant No. 20YF3WA009), and Cooperation Project Commissioned by Tanggula Animal Husbandry Development Co., LTD., Anduo County, Tibet Autonomous Region (Grand No. LIHPS2021 0077).

## Conflict of interest

The authors declare that the research was conducted in the absence of any commercial or financial relationships that could be construed as a potential conflict of interest.

## Publisher's note

All claims expressed in this article are solely those of the authors and do not necessarily represent those of their affiliated organizations, or those of the publisher, the editors and the reviewers. Any product that may be evaluated in this article, or claim that may be made by its manufacturer, is not guaranteed or endorsed by the publisher.
