# Supplementary material for: Enhancing activity of β-lactam and fluoroquinolones antibiotics by artemisinin and its derivatives against MDR Escherichia coli
Source: Front Vet Sci. 2022 Nov 10;9:1048531. doi: 10.3389/fvets.2022.1048531 (PMC9686389; doi:10.3389/fvets.2022.1048531)
Supplement: Supplementary file 1 [file Data_Sheet_1.DOCX]

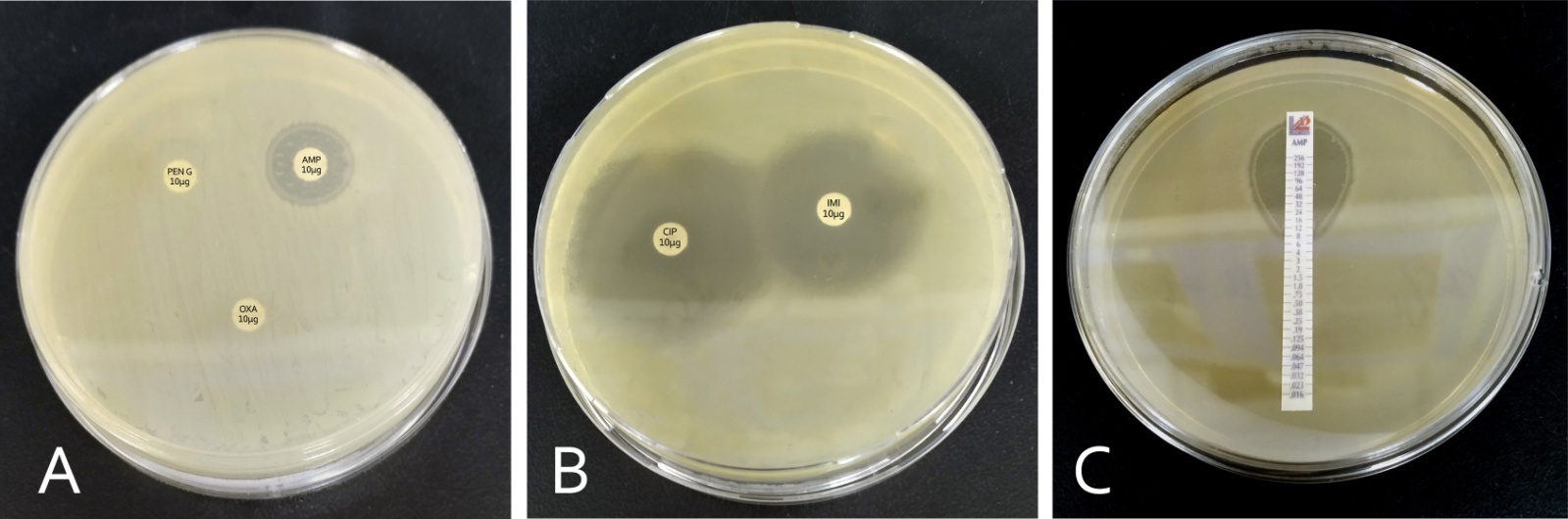


**Figure S1.** Disc diffusion test (A), (B) and E-strip test (C) of antibiotics against *E. coli*. Limited zones of inhibitions around most antibiotics showed multi-drug resistance


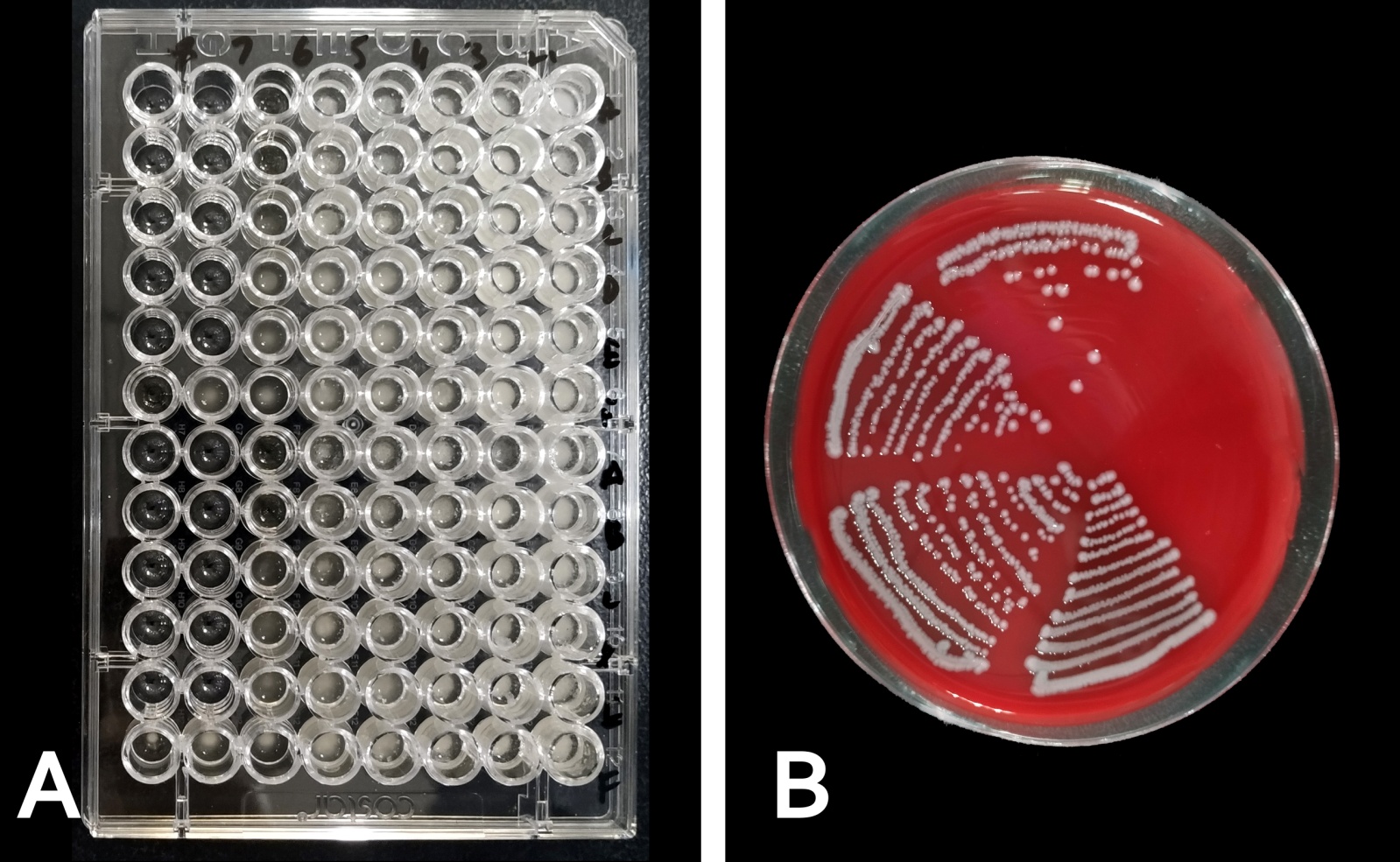


**Figure S2*.*** Test of synergism. Each vial in 96 well plate having different combinations of artemisinin and its derivatives with antibiotics. MDR *E. coli* were inoculated in these vials and incubated for 18 hours. After incubation results were determined by streaking the inoculums from vials to blood agar plates(plates were divided into six parts and having vials from a same row).These are the confirmatory tests from FICI. The vial showing growth from the 96 well plate means a limited combination dose of antibiotics and drug monomers. Vials showing no growth represent some synergistic or additive effects of the combinations.

*
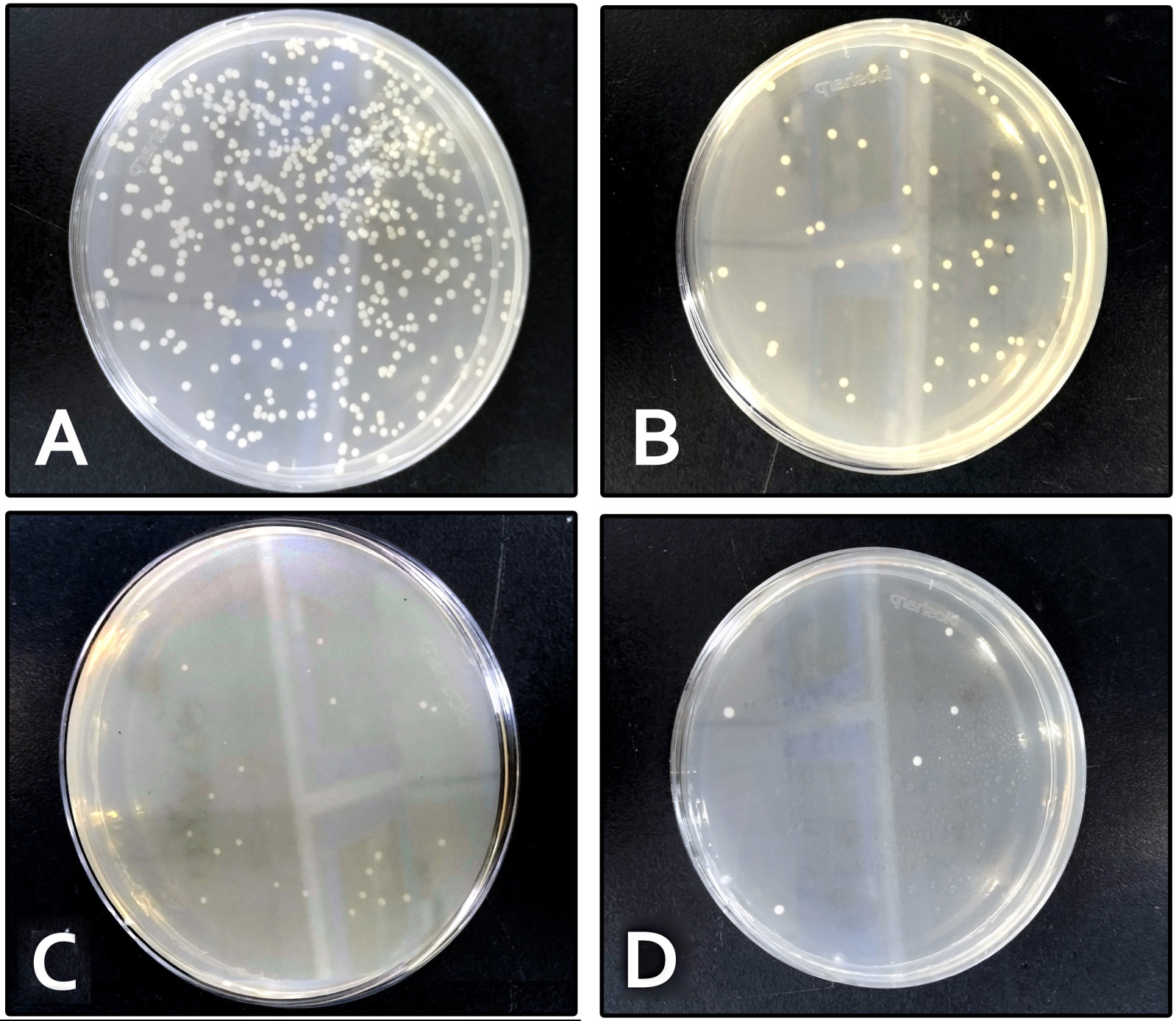
*

**Figure S3.** *E. coli* Colony counting of different time interval by usning Artemisinin and Penicillin G. At 8 hours, colony counting with varying dilutions of bacteria from a stock solution of 0.5 bacterial culture A= at 4 hours , B=6 hour , C=8 hours , D=16 hour.

***Figure S4.*** Comparison of drugs' minimum inhibitory concentrations (mg/mL) against *E. coli*.

***Figure S5.*** When used in combination, the percentage change in minimum inhibitory concentration (µg/mL) of antibiotics

***Figure 6.*** The percentage change in antibiotics' minimum inhibitory concentration (µg/mL) when used in combination.
