# Supplementary material for: Enhancing activity of β-lactam and fluoroquinolones antibiotics by artemisinin and its derivatives against MDR Escherichia coli
Source: Front Vet Sci. 2022 Nov 10;9:1048531. doi: 10.3389/fvets.2022.1048531 (PMC9686389; doi:10.3389/fvets.2022.1048531)
Supplement: Supplementary file 2 [file Table_1.DOCX]

***Table S1.*** Confirmation of antibiotic resistance profile of *E. coli* against different antibiotics using different tests*.*

| **Antibiotic names** | **E-test**  **(µg/mL)** | **ZOIs**  **(mm)** | **Broth microdilution method**  **MICs**  **(µg/mL)** |
| --- | --- | --- | --- |
| Penicillin | 172±88.63 | 1.245±1.376 | 215±251.147 |
| Ampicillin | 71.125±106.882 | 14.545±8.97 | 170.94±271.26 |
| Oxacillin | 256±0 | 0±0 | 360±174.36 |
| Ciprofloxacin | 0.017125±0.0022 | 37.12±1.596 | 0.0196±0.012 |
| Imipenem | 0.239±0.09262 | 29.035±1.317 | 0.625±0.383 |
